# More is not always better: longitudinal associations and nonlinear dose–response relationships between physical activity, psychological capital, and positive body image among Chinese university students

**DOI:** 10.3389/fpubh.2026.1870988

**Published:** 2026-08-28

**Authors:** Yi Li, Xi Chen

**Affiliations:** 1 School of Economics and Management, Guangzhou College of Applied Science and Technology, Guangzhou, China; 2 School of Sports Science, Guangzhou College of Applied Science and Technology, Guangzhou, China

**Keywords:** body appreciation, cross-lagged panel model, physical activity, positive body image, psychological capital, university students

## Abstract

**Objective:**

This study examined the longitudinal associations between physical activity (PA), psychological capital (PsyCap), and positive body image, operationalized as body appreciation, among Chinese university students. We further evaluated the mediating role of PsyCap and explored potential nonlinear dose–response relationships between different dimensions of PA and body appreciation.

**Methods:**

A two-wave longitudinal survey with a three-month interval was conducted among 669 university students from four universities in Z City, China. PA was assessed using the Physical Activity Rating Scale-3 (PARS-3), PsyCap was measured using the Psychological Capital Questionnaire-24 (PCQ-24), and body appreciation was assessed using the Body Appreciation Scale-2 (BAS-2). Cross-lagged panel models were used to examine prospective associations, longitudinal mediation was evaluated using bias-corrected bootstrap procedures, and polynomial regression models were fitted to investigate nonlinear dose–response relationships.

**Results:**

Male students reported significantly higher levels of PA, PsyCap, and body appreciation than female students (all *p* < 0.05). Baseline PA prospectively predicted higher PsyCap (*β* = 0.121, *p* < 0.001) and greater body appreciation (*β* = 0.264, *p* < 0.001) at follow-up, whereas baseline PsyCap also predicted subsequent body appreciation (*β* = 0.170, *p* < 0.001). Exercise intensity was linearly associated with PsyCap, whereas exercise duration and frequency exhibited U-shaped associations. For body appreciation, exercise intensity and frequency showed positive linear associations, whereas exercise duration demonstrated a significant cubic nonlinear association with body appreciation. Longitudinal mediation analysis indicated that PsyCap partially mediated the prospective association between baseline PA and follow-up body appreciation, accounting for 23.33% of the total association.

**Conclusion:**

Higher levels of PA were prospectively associated with greater PsyCap and body appreciation among Chinese university students. PsyCap partially accounted for the statistical covariation in the prospective association between PA and body appreciation. Different dimensions of PA demonstrated distinct dose–response patterns, suggesting that the psychological benefits of PA may vary according to exercise intensity, duration, and frequency. These findings indicate prospective temporal associations.

## 1 Introduction

### 1.1 Positive body image among university students

Positive body image is a multidimensional construct characterized by acceptance, appreciation, and respect for one’s body, extending beyond the mere absence of body dissatisfaction (1). In the present study, positive body image was operationalized as body appreciation, which was assessed using the Body Appreciation Scale-2 (BAS-2) (1). Body appreciation reflects positive attitudes toward one’s body, recognition of body functionality, and resistance to unrealistic sociocultural appearance ideals (2).

University students constitute an important population for research on positive body image because the transition to adulthood is accompanied by rapid identity development, heightened social comparison, and increased exposure to idealized appearance standards through both traditional and social media (3–5). Lower levels of body appreciation have consistently been associated with depression, anxiety, eating-related concerns, and poorer psychosocial functioning (6, 7).

These associations may also be shaped by cultural context. Compared with Western populations, body-related self-evaluation among Chinese university students is influenced not only by personal appearance ideals but also by collectivist values, interpersonal comparison, and concerns about maintaining social recognition (“face”) (8, 9). In collectivist cultures, body-related evaluations are more strongly shaped by perceived social expectations and interpersonal harmony, whereas in individualistic cultures they rely more heavily on personal appearance ideals. These cultural influences may affect how individuals develop and maintain body appreciation, underscoring the importance of examining positive body image within the Chinese sociocultural context rather than assuming that findings from Western populations are directly generalizable.

### 1.2 Physical activity and positive body image

A growing body of evidence indicates that physical activity (PA) is positively associated with positive body image. Regular participation in PA may enhance body appreciation by improving perceptions of physical competence, increasing awareness of body functionality, and promoting positive emotional experiences (10). Objectification theory provides one possible explanation for these associations by suggesting that PA may reduce appearance-based self-surveillance and encourage more adaptive body evaluations (11). Similarly, self-determination theory (12, 13) proposes that exercise motivated by intrinsic goals, such as health and enjoyment, is more likely to promote psychological wellbeing than exercise driven primarily by appearance concerns, a proposition supported by empirical research on exercise motivation and body image (14).

However, the psychological benefits of PA may depend not only on whether individuals engage in physical activity but also on how much and how often they participate. Insufficient PA may provide limited psychological stimulation (15), whereas excessive exercise may increase fatigue, exercise dependence, physical burden, or appearance-related pressure, thereby offsetting potential psychological benefits (15, 16). Consequently, different dimensions of PA—including exercise intensity, duration, and frequency—may exhibit distinct dose–response relationships with body appreciation rather than uniformly linear associations. The association between PA and body appreciation is therefore unlikely to follow a simple “more is always better” pattern. Previous studies have reported linear, U-shaped, inverted U-shaped, and S-shaped relationships between PA and psychological outcomes (17, 18). Nevertheless, evidence regarding these nonlinear dose–response relationships remains limited, particularly among Chinese university students (19), highlighting the need for longitudinal studies that simultaneously evaluate both temporal and nonlinear associations.

### 1.3 Physical activity, psychological capital, and the potential psychological pathway to positive body image

Psychological capital (PsyCap) is a positive psychological resource comprising self-efficacy, hope, optimism, and resilience (20, 21). In the PsyCap framework, optimism is conceptualized as a state-like, malleable positive expectation about future success, which is theoretically distinct from stable dispositional (trait) optimism (20). Individuals with higher PsyCap generally demonstrate greater confidence in overcoming challenges, maintaining motivation toward personal goals, and adapting to stressful situations. These characteristics have consistently been associated with better mental health, greater subjective wellbeing, and more adaptive coping across diverse populations (2, 22).

Participation in PA may contribute to the development of PsyCap through repeated experiences of goal attainment, mastery, emotional regulation, and successful adaptation to physical challenges (23, 24). Regular exercise has been associated with greater self-efficacy, resilience, optimism, and other positive psychological resources, suggesting that the benefits of PA extend beyond physical health alone (20, 23). Because these psychological resources are also associated with more adaptive self-evaluation and emotional wellbeing, PsyCap may represent an important psychological pathway linking PA with positive body image.

However, most available evidence has been derived from cross-sectional studies, making it difficult to establish the temporal ordering among PA, PsyCap, and body appreciation (25). Longitudinal evidence in university students remains limited, highlighting the need to examine whether changes in PsyCap prospectively explain the association between PA and body appreciation. Moreover, although alternative psychological mechanisms, such as physical self-perceptions (26), social physique anxiety (27), or exercise motivation (28), have been proposed to link PA with body image, PsyCap offers a distinct theoretical advantage: as a higher-order positive psychological resource, it captures the cumulative, cross-domain benefits of regular PA (e.g., mastery experiences, emotional regulation, goal pursuit) that transcend specific body-related perceptions (23). This meta-resource framework positions PsyCap as a particularly promising mechanism through which PA may foster not merely the absence of body dissatisfaction, but the active cultivation of positive body appreciation.

### 1.4 The potential mediating role of psychological capital

We propose that PsyCap represents a theoretically grounded and integrative mechanism that bridges the relationship between PA and positive body image. Unlike narrower domain-specific constructs such as body self-esteem or perceived physical competence that focus primarily on physical domain-specific evaluations, PsyCap represents a higher-order positive psychological resource encompassing four core dimensions: self-efficacy, hope, optimism, and resilience (21). This meta-resource is particularly well suited to explain how PA translates into body appreciation for three reasons. First, according to self-determination theory (12, 13), PA satisfies basic psychological needs for competence, autonomy, and relatedness, which in turn nourish the core components of PsyCap (14). Second, the broaden-and-build theory of positive emotions (29, 30) posits that recurrent positive experiences generated through PA expand individuals’ cognitive and behavioral repertoires, gradually building enduring psychological resources such as resilience and optimism, as elaborated in subsequent theoretical reviews (31). Third, objectification theory suggests that PA may reduce appearance-based self-surveillance; we extend this line of reasoning by proposing that the self-efficacy and resilience components of PsyCap empower individuals to resist sociocultural appearance pressures and reorient their self-evaluation toward body functionality and appreciation. Thus, by integrating complementary insights from self-determination theory (12, 13), objectification theory (32), and the broaden-and-build theory of positive emotions (29, 30), PsyCap offers a more comprehensive, multi-theoretical account of the pathways through which PA facilitates the development of positive body appreciation, as supported by empirical research on intrinsic motivation and body image (14, 31, 33).

From this integrative theoretical standpoint, individuals with higher PsyCap are posited to be less susceptible to appearance-driven social comparison and more likely to appreciate body functionality rather than fixating narrowly on physical appearance (34). This is because the self-efficacy and hope dimensions of PsyCap empower individuals to set and pursue intrinsic health goals, while optimism and resilience buffer against appearance-related setbacks and sociocultural pressures (35). Consequently, PsyCap may partially explain the prospective association between PA and body appreciation (25). It is important to note, however, that PsyCap is unlikely to be the sole pathway; alternative mechanisms—including changes in physical competence (35), social interaction (23), exercise motivation (36), and perceived health (19), may also contribute independently or synergistically to this association. Conceptualizing PsyCap as a partial mediator therefore reflects a theoretically conservative, methodologically realistic position that avoids overstating the scope of the proposed mechanism (37).

**Figure 1:**
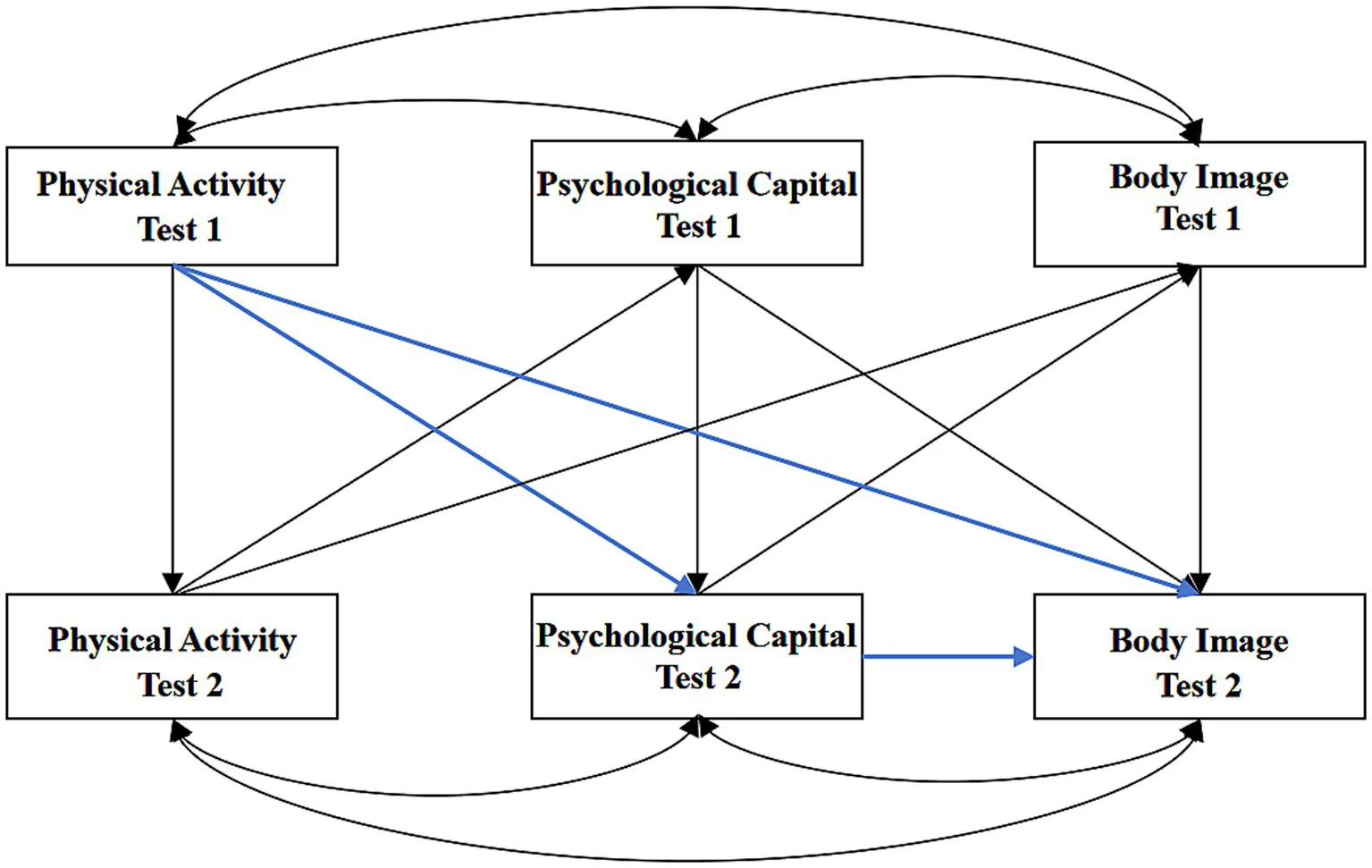
Assumption of cross-lag and mediation model.

Despite these robust theoretical foundations, critical empirical gaps substantially constrain current scholarly understanding of this relationship. First, existing research has most frequently examined PA, PsyCap, and body appreciation as disconnected, independent constructs, with the vast majority of published work relying exclusively on cross-sectional study designs. Consequently, the unambiguous temporal ordering of these interrelated variables—and specifically the fundamental question of whether PA antecedes PsyCap and subsequent body appreciation—remains empirically unconfirmed. Second, while multiple candidate psychological mechanisms have been proposed to explain the well-documented PA–body image association, no longitudinal study to date has formally tested whether PsyCap functions as a statistically significant mediator of this relationship across time. Third, nearly all prior research has treated PA as a unidimensional global exposure, implicitly assuming uniform linear associations between total PA volume and downstream psychological outcomes. This oversimplification overlooks the theoretically plausible possibility that distinct dimensions of PA (including intensity, duration, frequency) may exhibit heterogeneous dose–response relationships with psychological outcomes, including nonlinear patterns that have yet to be systematically mapped. Fourth, the majority of existing empirical evidence has been derived from Western, individualistic cultural contexts, leaving unaddressed the critical open question of whether these associations generalize to Chinese university students, whose body-related self-evaluations are shaped by cultural values, interpersonal appearance comparison, and culturally specific “face” concerns. To address these gaps, the present study integrates cross-lagged panel modeling, longitudinal mediation analysis, and polynomial regression within a two-wave prospective design. Specifically, we aim to (a) establish the temporal precedence of PA relative to PsyCap and body appreciation, (b) evaluate PsyCap as a hypothesized partial mediator of the prospective PA–body appreciation association between PA and body appreciation, and (c) identify distinct linear and nonlinear dose–response patterns between specific PA dimensions and both PsyCap and body appreciation. Given the inherent nature of this observational study design, any statistically significant mediating pathways identified must be interpreted as evidence of longitudinal statistical mediation, rather than definitive proof of a causal mechanism (Figure 1).

*Hypothesis 1*: Baseline physical activity levels prospectively predict subsequent psychological capital at follow-up.

*Hypothesis 2*: Baseline physical activity levels prospectively predict subsequent body appreciation at follow-up.

*Hypothesis 3*: Baseline psychological capital levels prospectively predict subsequent body appreciation at follow-up.

*Hypothesis 4*: Psychological capital partially mediates the prospective association between physical activity and body appreciation.

## 2 Materials and methods

### 2.1 Participants and study design

Sample size was estimated using G*Power3.1. In the absence of directly comparable longitudinal estimates, we adopted Cohen’s conventional medium effect sizes for behavioral research: *d* = 0.50 for the two-group comparison and *f*^2^ = 0.15 for the multiple-regression analyses (38). With a two-sided significance level of 0.05 and statistical power of 0.95, the minimum required sample sizes were 184 participants for the Wilcoxon–Mann–Whitney test and 107 participants for the cross-lagged panel and multiple-regression analyses. To ensure adequate power for the planned longitudinal and mediation analyses and to accommodate an anticipated attrition rate of 20%, recruitment exceeded these minimum requirements.

**Figure 2:**
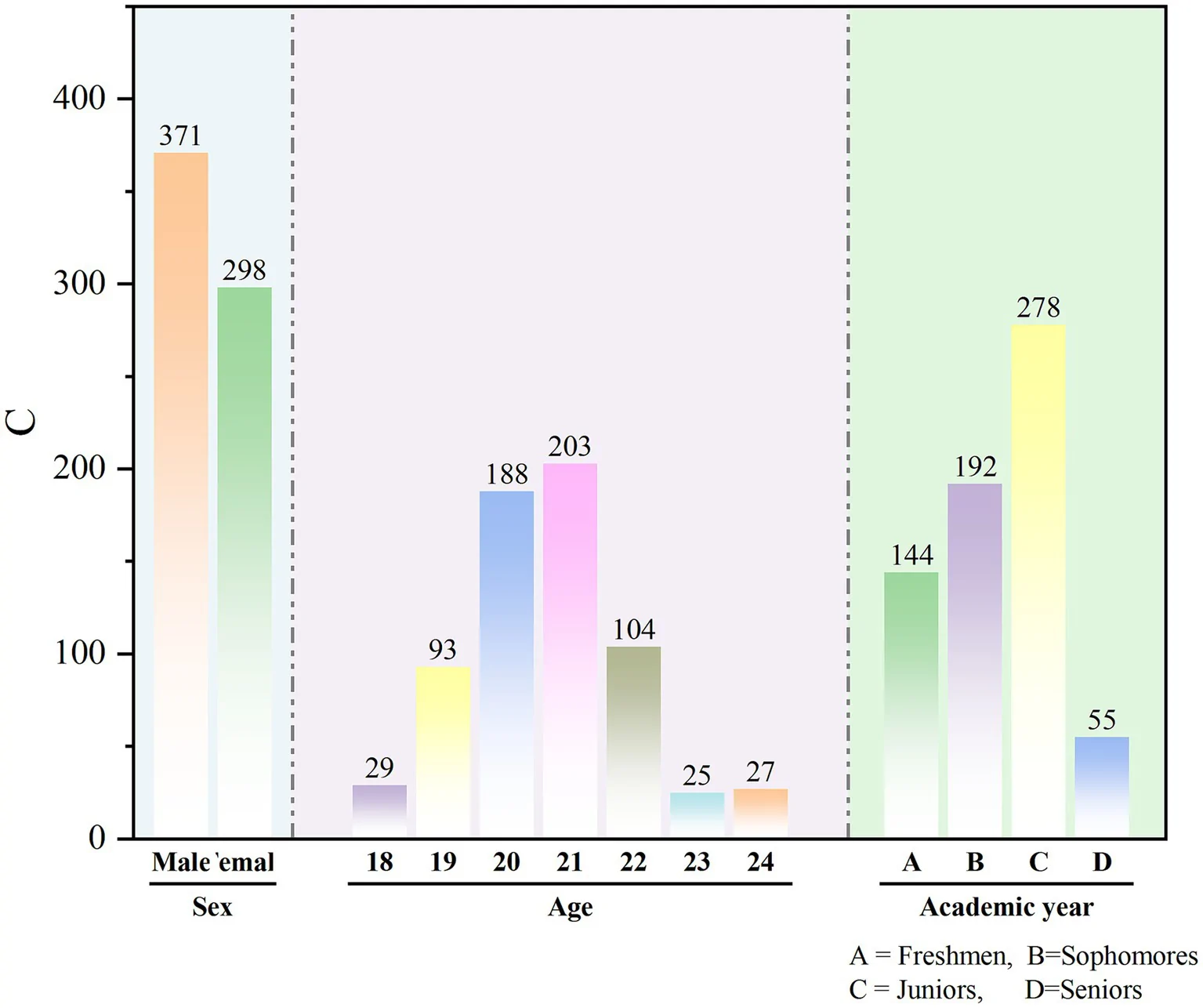
Participant flow through the two-wave longitudinal study. At Test 1, 759 questionnaires were distributed, and 741 valid questionnaires were obtained. At Test 2, 28 participants were lost to follow-up and 17 questionnaires were excluded because of invalid responses, resulting in 696 matched questionnaires. After excluding 27 questionnaires that did not meet the predefined eligibility criteria, 669 participants were included in the final statistical analyses. This same sample (*n* = 669) was used for all subsequent primary analyses—including sex-based comparisons, cross-lagged panel modeling, longitudinal mediation, and polynomial regression—unless explicitly stated otherwise.

A three-month, two-wave longitudinal survey was conducted using cluster sampling among students from four universities in Z City, China. The baseline survey (Test 1) was conducted from 3 to 9 September 2025 using the Questionnaire Star platform. The follow-up survey (Test 2) was conducted from 3 to 9 December 2025, yielding an inter-assessment interval of approximately 3 months (90 days). At baseline, 759 questionnaires were distributed, of which 741 were valid (97.62%). At follow-up, 696 matched questionnaires were obtained after excluding participants lost to follow-up and invalid questionnaires. Following application of the predefined eligibility criteria, the final analytical sample consisted of 669 participants (Figure 2).

Participants were eligible if they (1) were students enrolled at one of the four participating universities; (2) voluntarily participated in both survey waves; (3) were able to complete the questionnaires independently; and (4) provided valid responses at baseline. Participants were excluded if they had incomplete paired questionnaires, substantial missing data, duplicate submissions, obvious response patterns, logical inconsistencies, or failed to meet the predefined eligibility criteria.

### 2.2 Measures

#### 2.2.1 Physical activity

Physical activity was assessed using the Physical Activity Rating Scale-3 (PARS-3), originally developed by Liang (39). The PARS-3 has been widely used and validated for assessing physical activity in Chinese populations (40). The scale assesses three dimensions of PA, including exercise intensity, exercise duration, and exercise frequency. The total PA score was calculated as follows:

$$\begin{array}{l} \mathrm{PA}\;\text{score}=\text{Exercise Intensity}\times (\text{Exercise Duration}-1)\\ \times \text{Exercise Frequency} \end{array}$$

According to the recommended cut-off values, total scores of ≤19, 20–42, and ≥43 indicate low, moderate, and high levels of PA, respectively. Higher scores indicate higher levels of physical activity. Internal consistency was good, with Cronbach’s *α* values of 0.809 at Test 1 and 0.845 at Test 2.

#### 2.2.2 Body appreciation scale-2

Positive body image was operationalized as body appreciation and assessed using the Body Appreciation Scale-2 (BAS-2), originally developed by Tylka and Wood-Barcalow (1). The BAS-2 is a well-validated instrument specifically designed to assess body appreciation, which is widely regarded as a core dimension of positive body image. The scale comprises 10 items with a single-factor structure, each rated on a five-point Likert scale ranging from 1 (never) to 5 (always). The overall body appreciation score was calculated as the mean of the 10 items, with higher scores indicating greater body appreciation. The Chinese version of the BAS-2 has demonstrated satisfactory reliability and validity among Chinese adolescents and young adults (41). Internal consistency was excellent, with Cronbach’s *α* values of 0.951 at Test 1 and 0.957 at Test 2.

#### 2.2.3 Psychological capital questionnaire

Psychological capital was assessed using the Chinese version of the Psychological Capital Questionnaire (PCQ-24), translated and validated by Luthans et al. (21). The PCQ-24 comprises 24 items assessing four dimensions of PsyCap: self-efficacy, hope, resilience, and optimism, with six items for each dimension. All items are rated on a six-point Likert scale ranging from 1 (strongly disagree) to 6 (strongly agree). The overall PsyCap score was calculated as the mean of all 24 items, with higher scores indicating higher levels of psychological capital. The Chinese version of the PCQ-24 has demonstrated satisfactory reliability and validity in Chinese populations and has been widely used in previous studies (42). Internal consistency was excellent, with Cronbach’s *α* values of 0.946 at Test 1 and 0.952 at Test 2.

### 2.3 Ethical approval

This study was approved by the Academic Committee of Guangzhou College of Applied Science and Technology (Approval No. GZAST2025082901; approved on 29 August 2025). Oral informed consent was obtained from all participants before data collection. The use of oral rather than written informed consent was reviewed and approved by the Institutional Review Board. This approach was considered appropriate because the survey was anonymous, administered online, and involved minimal risk to participants. Participants were informed that participation was voluntary, that they could withdraw at any time without penalty, and that all collected data would remain confidential and be used exclusively for research purposes. All data were anonymized before analysis to ensure participant confidentiality. The study was conducted in accordance with the ethical principles of the Declaration of Helsinki.

### 2.4 Statistical analyses

Statistical analyses were performed using IBM SPSS Statistics 27.0, Mplus 8.0, and OriginLab 2021. Internal consistency was evaluated using Cronbach’s *α*, and common method bias was assessed using Harman’s single-factor test (43). Descriptive statistics were calculated for all study variables. Because the data did not fully satisfy the assumption of normality, the Mann–Whitney U test was used to compare differences between male and female participants, whereas the Wilcoxon signed-rank test was used to examine within-participant changes between Test 1 and Test 2. Multiple linear regression analyses were performed to examine the associations among the study variables. Polynomial regression models were fitted to evaluate potential nonlinear dose–response relationships between individual dimensions of physical activity and psychological capital or body appreciation. Cross-lagged panel models and longitudinal mediation analyses were conducted using Mplus 8.0. Sex, age, and academic year were included as covariates in the cross-lagged panel and longitudinal mediation models because these demographic characteristics may be associated with physical activity and the psychological outcomes examined in this study. Indirect effects were estimated using bias-corrected bootstrap confidence intervals based on 5,000 resamples, and mediation was considered statistically significant when the 95% confidence interval did not include zero. Model fit was evaluated using multiple goodness-of-fit indices, including the *χ*^2^/df ratio, Comparative Fit Index (CFI), Tucker–Lewis Index (TLI), Root Mean Square Error of Approximation (RMSEA), and Standardized Root Mean Square Residual (SRMR). All statistical tests were two-tailed, and *p* < 0.05 was considered statistically significant. All reported analyses were conducted using the final analytical sample of *n* = 669 participants.

## 3 Results

### 3.1 Common method variance

Harman’s single-factor test was performed to assess the presence of common method bias using all measurement items from the Physical Activity Rating Scale-3 (PARS-3), the Psychological Capital Questionnaire (PCQ-24), and the Body Appreciation Scale-2 (BAS-2). An unrotated exploratory factor analysis extracted seven factors with eigenvalues greater than 1. The first unrotated factor explained 39.82% of the total variance, which was below the commonly accepted threshold of 40% (43). These results indicate that common method bias was not a serious concern in the present study.

### 3.2 Distributions and between-wave comparisons

**Figure 3:**
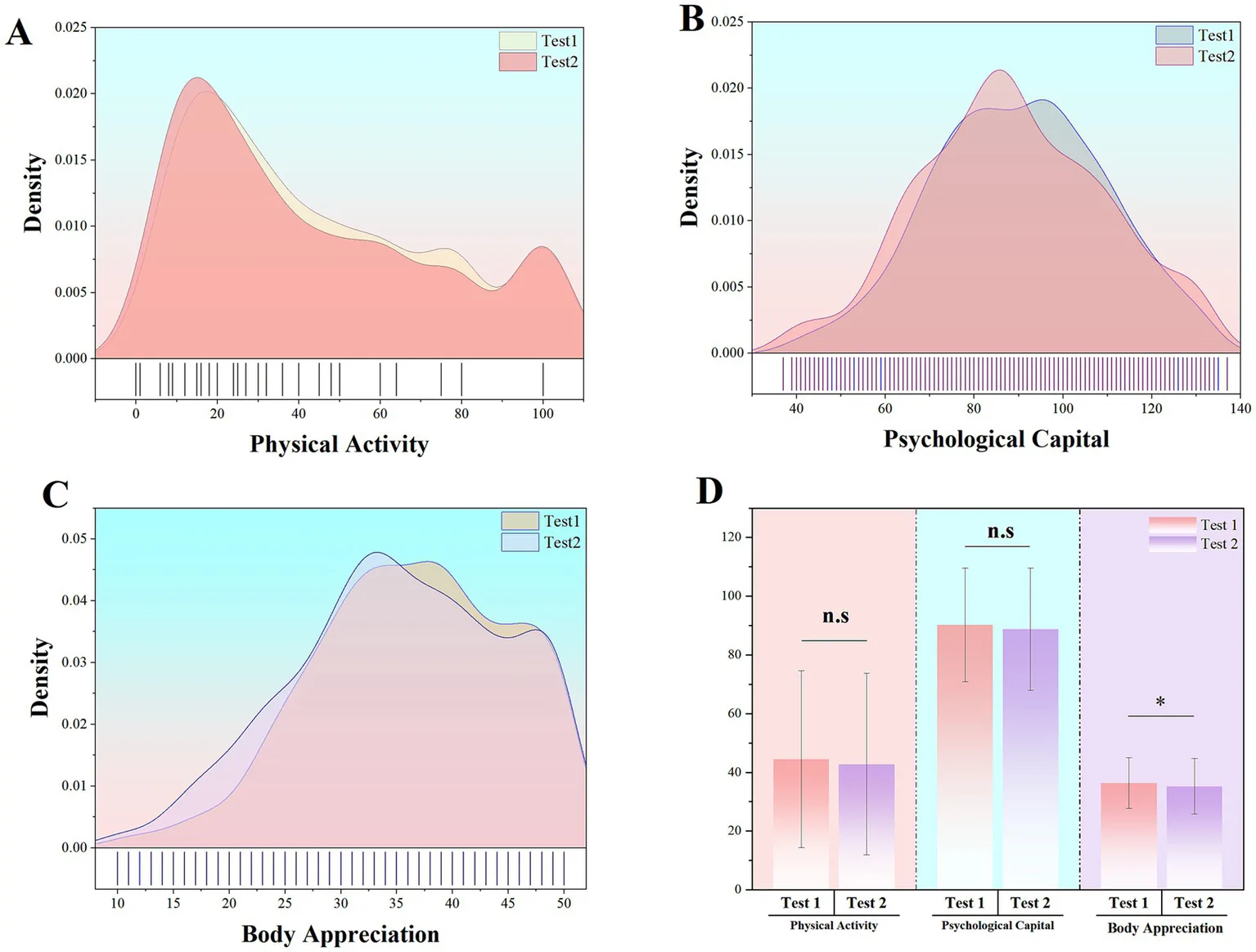
Distributions and between-wave comparisons of physical activity, psychological capital, and body appreciation at Test 1 and Test 2 (*n* = 669). Panels **(A–C)** show the distributions of physical activity, psychological capital, and body appreciation, respectively. Panel **(D)** presents between-wave comparisons as mean ± SD. Within-participant differences between Test 1 and Test 2 were evaluated using the Wilcoxon signed-rank test. n.s., not significant; *p < 0.05.

The distributions of PA, PsyCap, and body appreciation at Test 1 and Test 2 are presented in Figure 3. As shown in Figure 3A, the distributions of PA were broadly comparable across the two survey waves, with most participants scoring within the low-to-moderate PA range. Similarly, the distributions of PsyCap (Figure 3B) and body appreciation (Figure 3C) exhibited similar patterns at both assessments.

Between-wave comparisons are presented in Figure 3D. No significant differences were observed in PA or PsyCap between Test 1 and Test 2 (both *p* > 0.05). Specifically, the mean PA score changed from 44.2 ± 23.1 to 42.5 ± 21.8, whereas the mean PsyCap score changed from 89.3 ± 15.2 to 87.6 ± 17.1. In contrast, body appreciation showed a small but statistically significant decrease, declining from 36.1 ± 11.3 at Test 1 to 35.5 ± 10.7 at Test 2 (Wilcoxon signed-rank test: *Z* = −8.677, exact *p* < 0.001).

### 3.3 Sex-based descriptive statistics

#### 3.3.1 Comparisons between male and female participants

**Figure 4:**
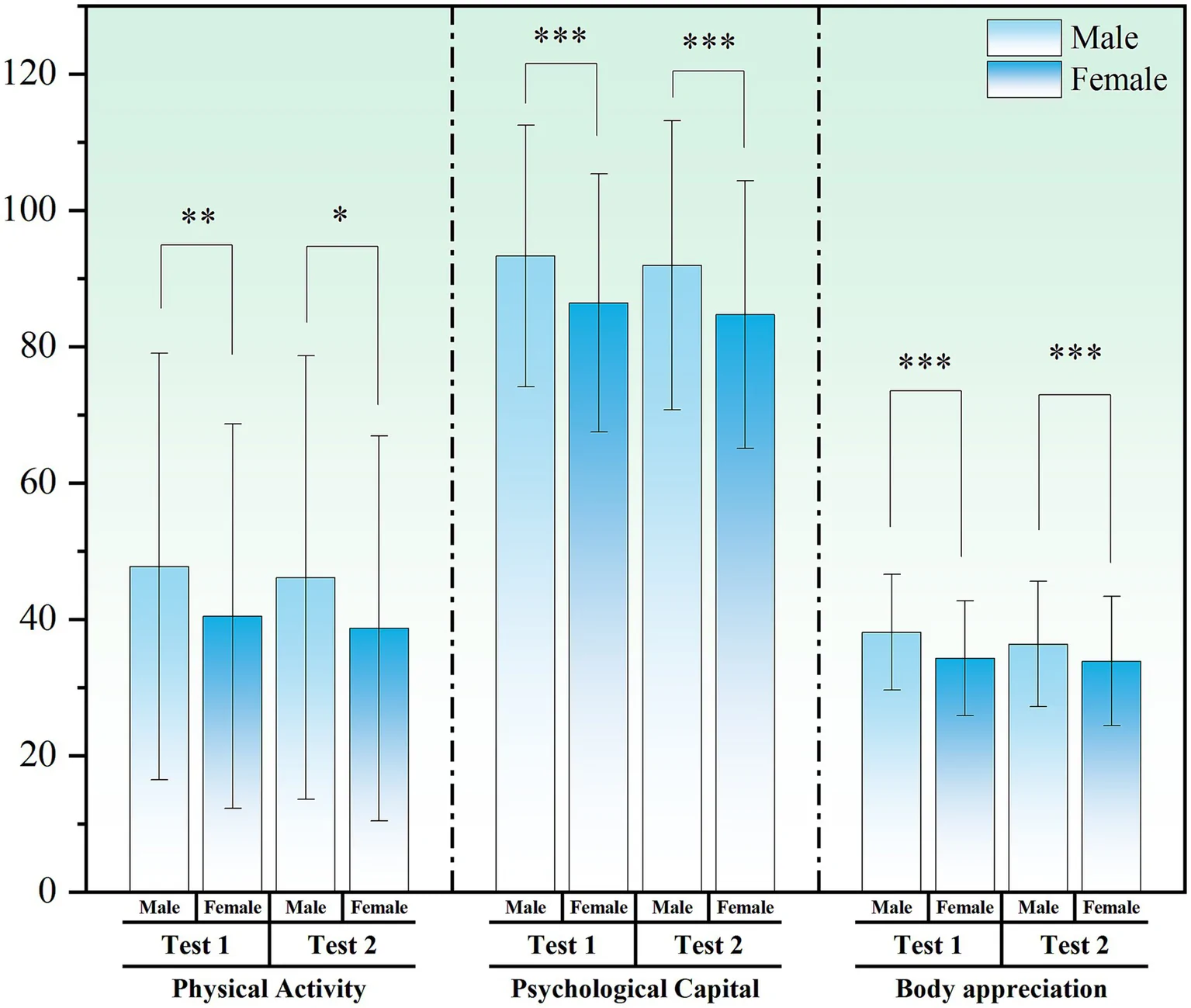
Sex differences in physical activity, psychological capital, and body appreciation. Values are presented as mean ± SD. *p < 0.05, **p < 0.01, and ***p < 0.001 (*n* = 669).

In the final analytical sample (*n* = 669), descriptive statistics for PA, PsyCap, and body appreciation by sex are presented in Figure 4. Male participants reported significantly higher PA than female participants at both Test 1 (47.76 ± 31.28 vs. 40.46 ± 28.32) and Test 2 (46.13 ± 32.53 vs. 38.66 ± 28.23) (*p* < 0.05; Supplementary Table S1).

Similarly, male participants demonstrated significantly higher PsyCap scores than female participants at both Test 1 (93.30 ± 19.19 vs. 86.43 ± 18.95) and Test 2 (91.96 ± 21.21 vs. 84.70 ± 19.65) (both *p* < 0.001; Supplementary Table S1).

Male participants also reported significantly higher body appreciation than female participants at Test 1 (38.11 ± 8.47 vs. 34.28 ± 8.43) and Test 2 (36.37 ± 9.21 vs. 33.87 ± 9.47) (both *p* < 0.001; Supplementary Table S1).

#### 3.3.2 Age distribution of study variables

Age-related patterns in PA, PsyCap, and body appreciation were also examined. PA showed only modest age-related variation, with relatively lower values among participants aged 20–22 years and a slight increase in the 23–24-year-old age group. Body appreciation demonstrated remarkable stability across all age cohorts, with only a slight increase observed in older participants. Similarly, PsyCap demonstrated minimal age-related variation across the age spectrum, and the longitudinal trajectories were highly comparable between Test 1 and Test 2 (see Supplementary Figure S1).

### 3.4 Correlations among study variables

**Figure 5:**
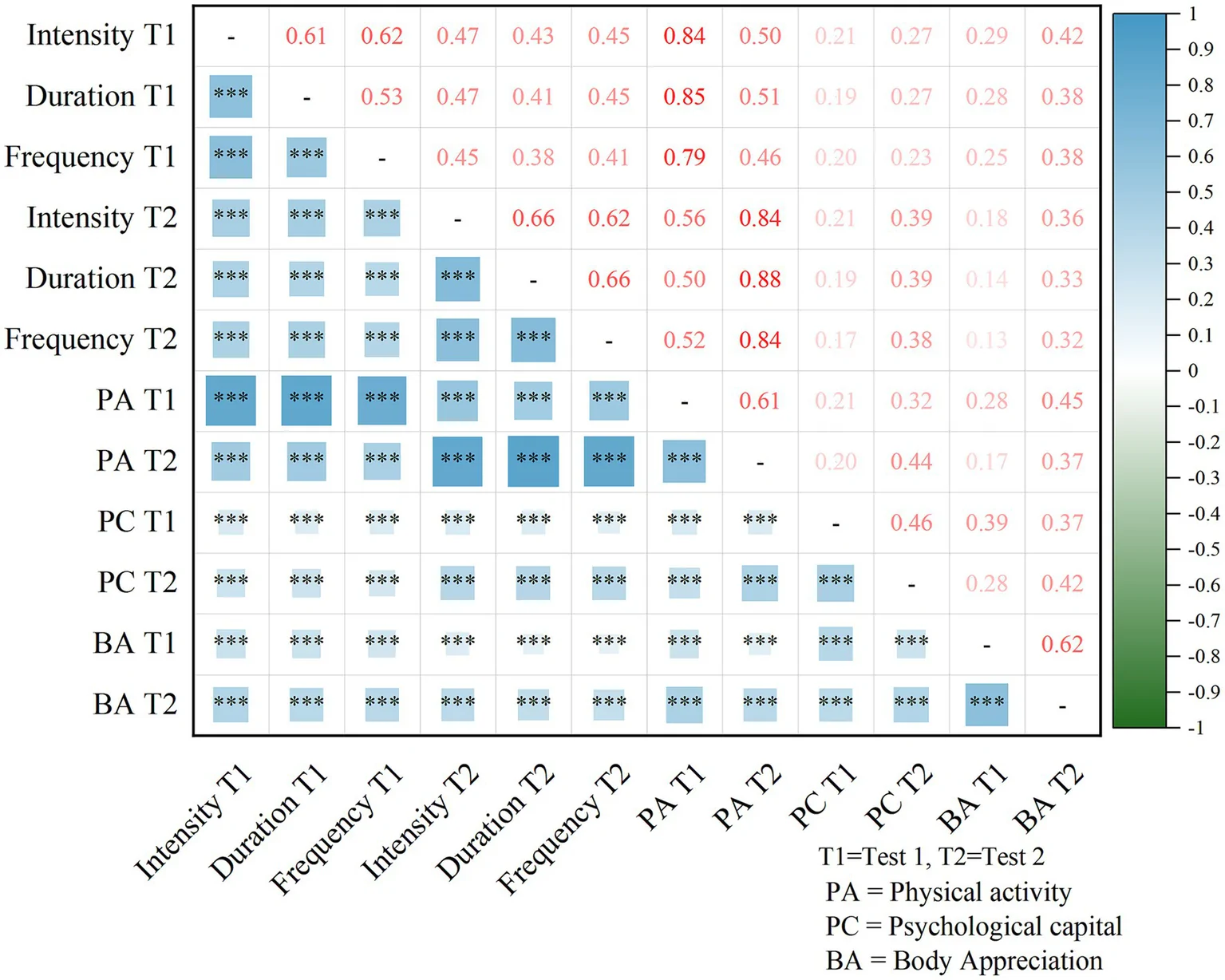
Correlation matrix of physical activity, psychological capital, and body appreciation at Test 1 and Test 2 (*n* = 669). Pearson correlation coefficients are shown in the upper triangle. Statistical significance is indicated in the lower triangle. *p < 0.05, **p < 0.01, and ***p < 0.001.

Pearson correlation coefficients among the study variables at Test 1 and Test 2 are presented in Figure 5. Significant positive correlations were observed both within and across the two measurement occasions. The three dimensions of physical activity (exercise intensity, exercise duration, and exercise frequency) were strongly correlated with the overall PA score at both Test 1 and Test 2 (*r* = 0.79–0.88, all *p* < 0.001). In addition, PA was positively correlated with PsyCap and body appreciation at both time points (*r* = 0.17–0.44, all *p* < 0.001). PsyCap was also positively associated with body appreciation at Test 1 and Test 2 (*r* = 0.28–0.42, all *p* < 0.001). Furthermore, all three major constructs demonstrated moderate temporal stability across the two survey waves, with significant correlations between Test 1 and Test 2 (PA: *r* = 0.61; PsyCap: *r* = 0.46; body appreciation: *r* = 0.62; all *p* < 0.001). These findings support the subsequent cross-lagged panel and longitudinal mediation analyses.

### 3.5 Cross-lagged panel analysis

Using the final analytical sample (*n* = 669), cross-lagged panel models (CLPMs) were estimated using Mplus 8.0 to examine the prospective associations among PA, PsyCap, and body appreciation across the two survey waves while controlling for sex, age, and academic year. To evaluate the longitudinal stability of these associations, four progressively constrained CLPMs were compared. Model 1 served as the unconstrained baseline model, whereas Models 2–4 successively imposed equality constraints on the cross-lagged paths, autoregressive paths, or both. As shown in Table 1, among the four competing models, Model 2 demonstrated the best balance between model fit and parsimony and was therefore selected as the final model for subsequent analyses (44). The selected model showed an acceptable fit to the data (*χ*^2^ = 16.083, df = 5, CFI = 0.991, TLI = 0.942, SRMR = 0.019, RMSEA = 0.058).

**Table 1:** Model fits and comparisons for cross-lagged panel models (*n* = 669).

Models	*χ* ^2^	df	CFI	TLI	SRMR	RMSEA	95% CI for RMSEA		AIC	BIC
							Lower	Upper		
Model 1	0^***^	0	1.000	1.000	0.000	0.000	0.000	0.000	37023.068	37266.380
Model 2	16.083^***^	5	0.991	0.942	0.019	0.058	0.028	0.090	37029.150	37249.934
Model 3	15.630^***^	2	0.989	0.823	0.030	0.101	0.058	0.150	37034.697	37268.998
Model 4	416.724^***^	8	0.678	−0.329	0.096	0.276	0.254	0.299	37423.791	37631.057

Model 1 = unconstrained (saturated) baseline model; Model 2 = Cross-lagged paths were constrained to equality across time; Model 3: Autoregressive paths were constrained to equality across time; Model 4: Both cross-lagged and autoregressive paths were constrained to equality across time. *χ*^2^, Chi-Square Value; df, Degrees of Freedom; ***p < 0.001.

**Figure 6:**
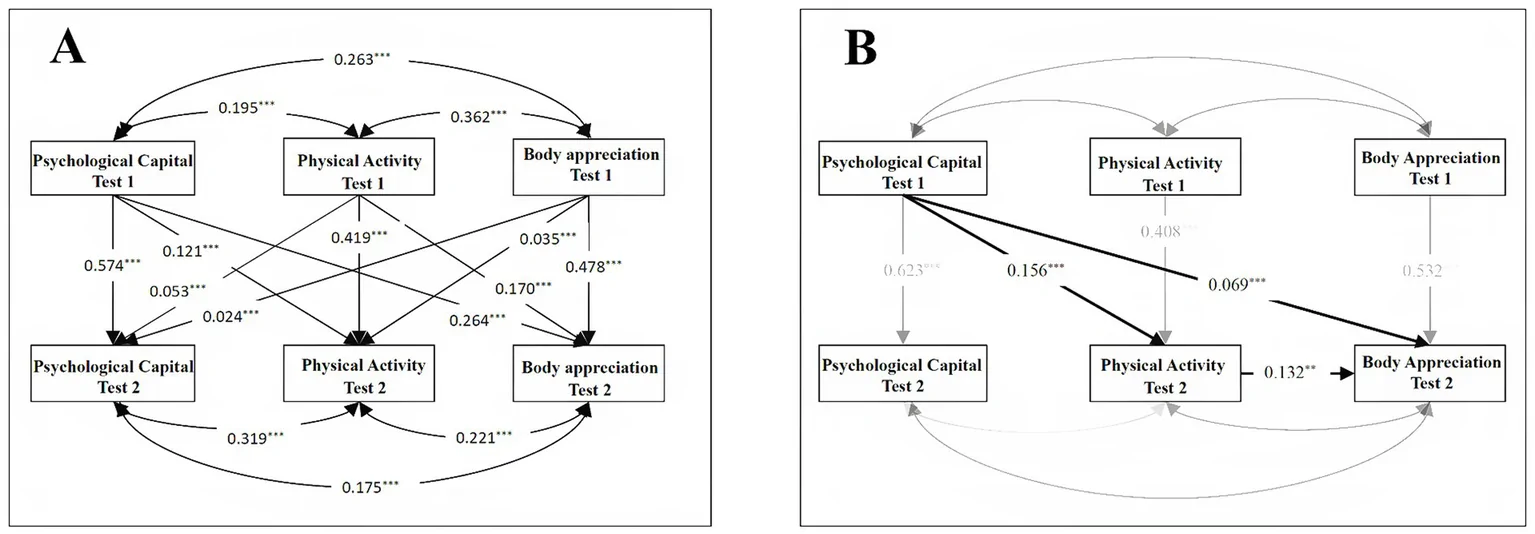
**(A)** Cross-lagged panel model of physical activity, psychological capital, and body appreciation; **(B)** Longitudinal mediation model of the association between physical activity, psychological capital, and body appreciation. *p < 0.05, **p < 0.01, and ***p < 0.001 (*n* = 669).

As illustrated in Figure 6A, baseline PA significantly and positively predicted PsyCap at Test 2 (*β* = 0.121, *p* < 0.001) and body appreciation at Test 2 (*β* = 0.264, *p* < 0.001). Baseline PsyCap also significantly predicted body appreciation at Test 2 (*β* = 0.170, *p* < 0.001) and PA at Test 2 (*β* = 0.053, *p* < 0.001). The baseline BA significantly and positively predicted PsyCap at Test 2 (*β* = 0.035, *p* = 0.004) and PA at Test 2 (*β* = 0.024, *p* < 0.001).

Bootstrap analyses based on 5,000 resamples indicated that the 95% confidence intervals for all significant cross-lagged paths excluded zero (Table 2), supporting the robustness of these prospective associations (45). Overall, baseline PA prospectively predicted subsequent PsyCap and body appreciation, whereas baseline PsyCap prospectively predicted subsequent body appreciation (46). Importantly, the cross-lagged model also revealed significant reverse-direction prospective associations: baseline PsyCap predicted subsequent PA (*β* = 0.053, *p* < 0.001), baseline body appreciation predicted subsequent PA (*β* = 0.024, *p* < 0.001), and baseline body appreciation predicted subsequent PsyCap (*β* = 0.035, *p* = 0.004). These reverse pathways were not pre-specified in our hypotheses and should be treated as exploratory post-hoc findings. While these additional associations carry modest theoretical relevance, their small effect sizes (*β* = 0.024--0.053) necessitate cautious interpretation, and they do not hold equivalent theoretical standing to the *a priori* hypothesized PA-led pathways. First, while the hypothesized path from PA to PsyCap was supported (*β* = 0.121), the unhypothesized reverse path from PsyCap to PA was also significant (*β* = 0.053). However, given that the hypothesized PA → PsyCap path was more than twice the magnitude of the reverse path, and considering the very small effect size of the reverse path, PA should be interpreted as the primary temporal antecedent in this relationship. The PsyCap → PA path instead represents a minor, exploratory reciprocal association that requires independent replication in multi-wave or experimental designs before any formal interpretations of dynamic or feedback interpretations can be advanced. Second, the finding that baseline body appreciation prospectively predicted both PA and PsyCap tentatively extends positive body image literature by suggesting that body appreciation is not merely a passive outcome of health behaviors but may to a limited degree also function as an active psychological resource that fosters subsequent health-promoting behaviors and positive psychological development. Third, the presence of significant cross-lagged paths in both directions does not constitute evidence for a dynamic or interconnected system. Because the three reverse paths were not pre-specified in our hypotheses and their standardized coefficients (*β* = 0.024–0.053) were substantially smaller than those of the hypothesized forward paths (*β* = 0.121–0.264), these findings do not establish meaningful bidirectional influences. Rather, they suggest that while PA functions as the primary temporal antecedent, small reverse associations may exist; however, given their very small effect sizes, these should be treated as exploratory findings requiring independent confirmation in multi-wave or experimental designs before any systems-based interpretations can be made. This pattern of mutual influence offers tentative, small-effect support for more dynamic, systems-based conceptualizations of health behavior and psychological wellbeing, but should not be interpreted as strong empirical validation of such frameworks.

**Table 2:** Standardized path coefficients and 95% confidence intervals for the cross-lagged panel model (*n* = 669).

Path	Estimate	S.E.	*p*	95% CI	
				Lower	Upper
PA (Test 1) → PsyCap (Test 2)	0.121	0.013	<0.001	0.095	0.147
PsyCap (Test 1) → PA (Test 2)	0.053	0.006	<0.001	0.041	0.064
PA (Test 1) → BA (Test 2)	0.264	0.028	<0.001	0.210	0.319
BA (Test 2) → PA (Test 1)	0.024	0.003	<0.001	0.018	0.029
PsyCap (Test 1) → BA (Test 2)	0.170	0.018	<0.001	0.135	0.205
BA (Test 1) → PsyCap (Test 2)	0.035	0.004	0.004	0.027	0.042

### 3.6 Associations between dimensions of physical activity and psychological capital

#### 3.6.1 Associations between physical activity dimensions and psychological capital

**Figure 7:**
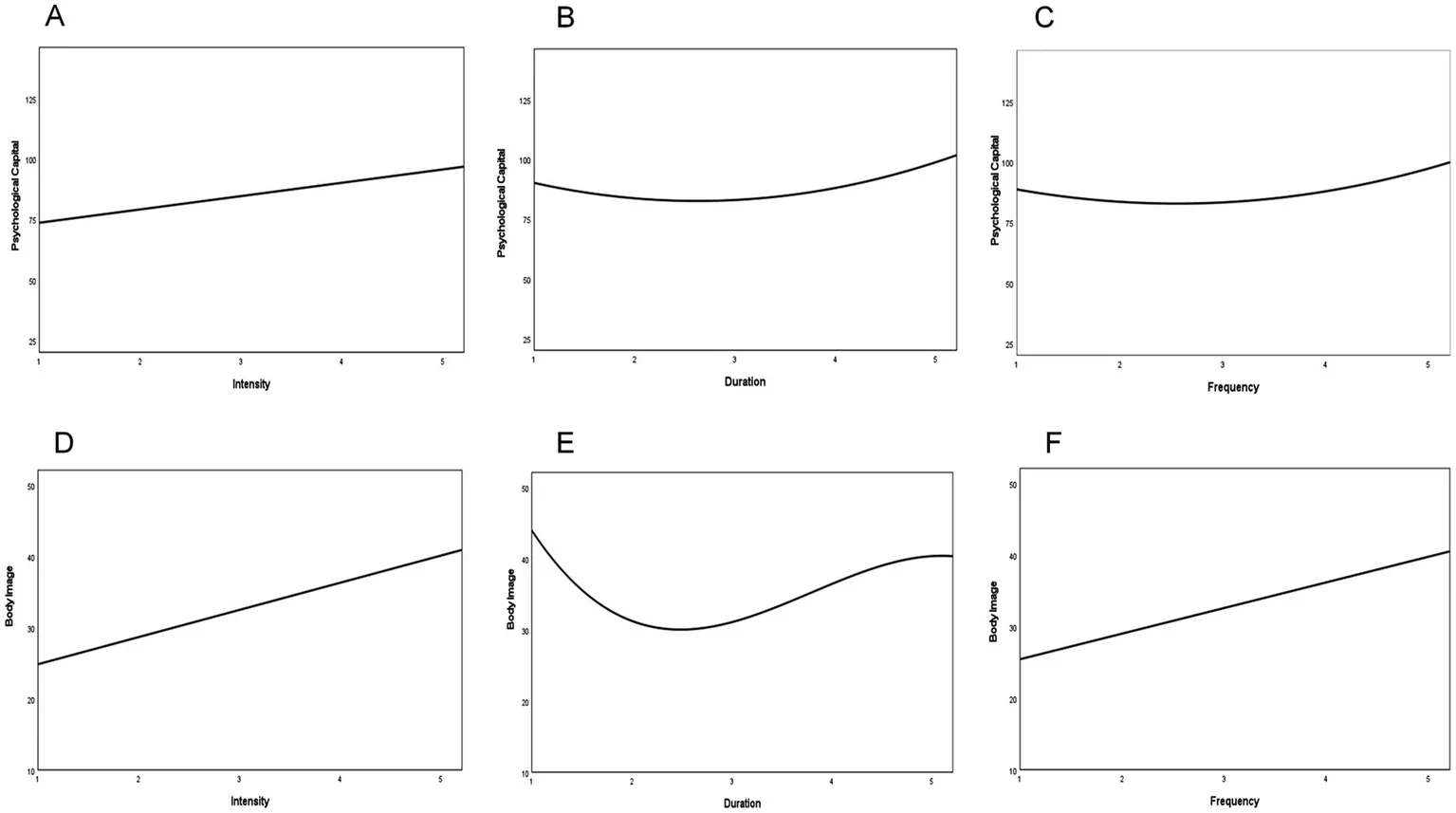
Linear and nonlinear associations between physical activity dimensions and psychological capital **(A–C)** and body appreciation **(D–F)** (*n* = 669).

In the full analytical sample (*n* = 669), regression analyses were conducted to examine the associations between different dimensions of PA and PsyCap. The results are presented in Table 3 and Figures 7A–C. Prior to regression analyses, diagnostic tests were conducted to assess model assumptions. Variance inflation factors (VIFs) for all predictors were below the commonly accepted threshold, indicating no evidence of problematic multicollinearity. The normality of residuals was evaluated using the Shapiro–Wilk test and normal Q–Q plots. Detailed diagnostic results are presented in Supplementary Table S2 and Supplementary Figures S2–S4.

**Table 3:** Regression coefficients for the associations between physical activity dimensions and psychological capital (*n* = 669).

Physical activity‌	*R* ^2^	△*R*^2^	*F*	*B*	*t*	*p*	VIF	B 95% CI	
								Lower	Upper
Intensity	0.074	0.073	53.518^***^	0.273	7.316	<0.001	1.000	0.199	0.346
Duration	0.089	0.086	32.383^***^	0.283	7.635	<0.001	1.005	0.210	0.356
Duration**2				0.108	3.090	0.002	1.005	0.039	0.176
Frequency	0.071	0.068	25.539^***^	0.277	7.006	<0.001	1.120	0.199	0.355
Frequency**2				0.117	3.632	<0.001	1.120	0.054	0.180

Linear and polynomial regression models were selected according to the best-fitting relationship between each physical activity dimension and psychological capital. **p < 0.01, ***p < 0.001.

Exercise intensity showed a significant positive linear association with PsyCap (*B* = 0.273, *p* < 0.001; *R*^2^ = 0.074) (Figure 7A), indicating that higher exercise intensity was associated with higher PsyCap. However, the model explained only 7.4% of the variance in PsyCap (*R*^2^ = 0.074), suggesting that while the association between exercise intensity and PsyCap was statistically significant, its practical or clinical significance in isolation is likely modest. This result suggests that a substantial proportion of the variability in PsyCap remains attributable to other factors not included in the present model.

Exercise duration demonstrated a significant U-shaped nonlinear association with PsyCap (*R*^2^ = 0.089, *F* = 32.383, *p* < 0.001). The linear term was significantly negative (*B* = 0.283, *p* < 0.001), whereas the quadratic term was significantly positive (*B* = 0.108, *p* = 0.002), indicating that PsyCap initially decreased and subsequently increased as exercise duration increased (Figure 7B). Nevertheless, the model accounted for only 8.9% of the variance in PsyCap (*R*^2^ = 0.089), indicating that while the identified nonlinear pattern is statistically robust, it explains a relatively modest proportion of the total variability in PsyCap, suggesting that other unmeasured factors likely play a more substantial role in shaping this psychological resource.

Similarly, exercise frequency exhibited a significant U-shaped nonlinear association with PsyCap (*R*^2^ = 0.071, *F* = 25.539, *p* < 0.001). The linear coefficient was significantly negative (*B* = 0.277, *p* < 0.001), whereas the quadratic coefficient was significantly positive (*B* = 0.117, *p* < 0.001), indicating that PsyCap initially decreased and subsequently increased with increasing exercise frequency (Figure 7C). Again, the explained variance was modest (7.1%), suggesting that exercise frequency is only one of many contributors to PsyCap and that its practical impact should be interpreted cautiously.

#### 3.6.2 Associations between physical activity dimensions and body appreciation

In the same analytical sample (*n* = 669), regression analyses were conducted to examine the associations between different dimensions of PA and body appreciation. The results are presented in Table 4 and Figure 7D–F. Prior to regression analyses, diagnostic tests were conducted to assess model assumptions. Variance inflation factors (VIFs) for all predictors indicated no evidence of problematic multicollinearity. Although the Shapiro–Wilk test suggested deviations from normality for some models, the normal Q–Q plots showed acceptable residual distributions. Considering the relatively large sample size, the regression models were considered appropriate for interpretation. Detailed diagnostic results are presented in Supplementary Table S2 and Supplementary Figures S5–S7.

**Table 4:** Regression coefficients for the associations between physical activity dimensions and body appreciation (*n* = 669).

Physical activity‌	*R* ^2^	△*R*^2^	*F*	*B*	*t*	*p*	VIF	B 95% CI	
								Lower	Upper
Intensity	0.174	0.173	140.405^***^	0.417	11.849	<0.001	1.000	0.348	0.486
Duration	0.160	0.156	42.116^***^	0.426	0.442	0.002	1.351	0.351	0.522
Duration**2				0.932	0.326	0.002	1.351	0.835	0.994
Duration**3				0.478	0.295	0.002	1.351	0.326	0.524
Frequency	0.145	0.144	112.988^***^	0.381	10.630	<0.001	1.000	0.310	0.451

The best-fitting linear or polynomial regression model was selected for each physical activity dimension based on model fit. **p < 0.01, ***p < 0.001.

Exercise intensity showed a significant positive linear association with body appreciation (*B* = 0.417, *p* < 0.001; *R*^2^ = 0.174) (Figure 7D), indicating that higher exercise intensity was associated with greater body appreciation. The model explained 17.4% of the variance in body appreciation (*R*^2^ = 0.174), which, although larger than that observed for PsyCap, still indicates that the majority of variability in body appreciation is attributable to factors beyond exercise intensity alone.

Exercise duration demonstrated a significant nonlinear S-shaped association with body appreciation (*R*^2^ = 0.160, *F* = 42.116, *p* < 0.001). The linear (*B* = 0.426, *p* = 0.002), quadratic (*B* = 0.932, *p* = 0.002), and cubic (*B* = 0.478, *p* = 0.002) terms were all statistically significant, indicating a complex nonlinear relationship between exercise duration and body appreciation (Figure 7E). However, the model accounted for only 16.0% of the variance, suggesting that while the cubic pattern is statistically detectable, its practical utility for predicting body appreciation is limited.

Exercise frequency also showed a significant positive linear association with body appreciation (*B* = 0.381, *p* < 0.001; *R*^2^ = 0.145) (Figure 7F), indicating that higher exercise frequency was associated with greater body appreciation. The explained variance was 14.5%, again underscoring that exercise frequency is a statistically significant yet practically modest predictor of body appreciation.

### 3.7 Longitudinal mediation analysis

A longitudinal mediation model was constructed using Mplus 8.0 in the final sample of 669 participants to examine whether PsyCap statistically mediated the prospective association between baseline PA and subsequent body appreciation while controlling for sex, age, and academic year. The model demonstrated an acceptable fit to the data (*χ*^2^/df = 9.924, RMSEA = 0.059, GFI = 0.995, TLI = 0.940, SRMR = 0.013) (Table 5).

**Table 5:** Fit indices of the longitudinal mediation model (*n* = 669).

Mediation model	
*χ^2^ (df)*	9.924^*^ (3)
RMSEA	0.059
CFI	0.995
TLI	0.940
SRMR	0.013

^*^p < 0.05.

As shown in Figure 6B, baseline PA significantly predicted PsyCap at Test 2 (*β* = 0.156, p < 0.001) and body appreciation at Test 2 (*β* = 0.069, *p* < 0.001). In addition, PsyCap at Test 2 was positively associated with body appreciation at Test 2 (*β* = 0.132, *p* = 0.010).

Bootstrap analyses based on 5,000 resamples indicated that the standardized indirect effect of PA on body appreciation through PsyCap was statistically significant [*β* = 0.021, 95% CI (0.006, 0.043)] and did not include zero (Table 6), indicating a significant partial mediation effect (45). The indirect effect accounted for 23.333% of the total effect, whereas the direct effect accounted for 76.667% of the total effect. These findings suggest that PsyCap partially mediated the prospective association between PA and body appreciation (47). Therefore, Hypothesis 4 was supported.

**Table 6:** Direct, indirect, and total effects of the longitudinal mediation model (*n* = 669).

Path	Standardized estimate	*SE*	*p*	95% *CI*		Relative effect (%)
				Lower	Upper	
Direct effect	0.069	0.016	<0.001	0.035	0.100	76.667
Indirect effect	0.021	0.010	0.030	0.006	0.043	23.333
Total effect	0.090	0.012	<0.001	0.066	0.113	

## 4 Discussion‌

The present two-wave longitudinal study investigated the prospective associations between PA, PsyCap, and positive body image (operationalized as body appreciation) among Chinese university students, while further exploring the potential nonlinear dose–response relationships between different dimensions of PA and the two psychological outcomes. The study makes four specific theoretical contributions. First, by demonstrating that baseline PA prospectively predicted greater PsyCap and body appreciation at follow-up, we provide longitudinal evidence supporting the temporal priority of PA as a plausible antecedent rather than merely a correlate of these positive psychological outcomes at the group level. Although the exploratory reverse cross-lagged paths were also statistically significant, their small effect sizes and unhypothesized post-hoc nature mean that the hypothesized PA-led prospective model remains the primary interpretive framework. However, because the conventional CLPM inherently conflates between-person differences with dynamic within-person processes, these findings cannot establish that an individual’s increase in PA directly generates a corresponding individual-level rise in PsyCap or body appreciation. Instead, they merely indicate that individuals with higher baseline PA tend to report higher subsequent levels of these outcomes variables, on average. This finding advances existing cross-sectional literature by clarifying the directional nature of these associations in longitudinal, group-level data. Second, by identifying PsyCap as a statistically significant partial mediator, we propose and empirically substantiate an integrative theoretical mechanism that underlying process linking PA to body appreciation. Specifically, our findings indicate that baseline PA is prospectively associated with higher levels of generalizable psychological resources (self-efficacy, hope, optimism, and resilience), which are in turn prospectively associated with more adaptive, functionality-oriented body evaluations. These observed temporal patterns align closely with the theoretical proposition that PA fosters the development of psychological resources that facilitate the formation of positive body image, though they do not provide definitive causal proof for this claim. This extends prior research that has focused on domain-specific mediators (e.g., physical self-worth) by highlighting the explanatory role of a higher-order meta-resource. Third, by disaggregating PA into its constituent dimensions and revealing distinct linear and nonlinear dose–response patterns, we challenge the prevailing “more PA is always better” assumption and demonstrate that the psychological benefits of PA are dimension-specific. This contributes to a more nuanced theoretical understanding of how different exercise characteristics independently and interactively shape psychological outcomes. Fourth, by replicating and extending these associations within a Chinese sociocultural context, we provide preliminary evidence that the prospective links among PA, PsyCap, and body appreciation are not confined to Western, individualistic populations, thereby enhancing the cross-cultural generalizability of positive body image theory. It is critical to reiterate that these contributions reflect temporal patterns observed in observational data; and they do not establish definitive causal effects of PA on PsyCap or body appreciation, nor do they confirm the causal priority of any specific mediational pathway. Three principal findings emerged. First, higher baseline PA prospectively predicted greater PsyCap and body appreciation at follow-up. Second, PsyCap partially mediated the prospective association between PA and body appreciation; however, the magnitude of this indirect effect was relatively modest, suggesting that PsyCap represents only one of several potential pathways linking PA with body appreciation. Third, different dimensions of PA demonstrated distinct dose–response patterns, with exercise intensity showing predominantly linear associations, whereas exercise duration and frequency exhibited nonlinear relationships with PsyCap and body appreciation. Finally, these findings extend previous evidence derived primarily from cross-sectional studies by providing longitudinal evidence for the temporal associations among PA, PsyCap, and positive body image among Chinese university students.

The present study found that baseline PA prospectively predicted higher PsyCap at follow-up, supporting Hypothesis 1 and extending previous evidence derived predominantly from cross-sectional studies (23). This finding is consistent with earlier research showing that regular participation in physical activity is associated with greater self-efficacy, optimism, hope, and resilience, which collectively constitute the core components of PsyCap (23). Theoretically, self-determination theory (12, 13) posits that engagement in PA provides repeated opportunities to experience competence, autonomy, and mastery, which may facilitate the accumulation of positive psychological resources (14, 33). Similarly, the broaden-and-build theory suggests that positive experiences associated with physical activity may broaden individuals’ cognitive and behavioral repertoires, potentially building enduring psychological resources such as resilience and optimism (30). Together, these theoretical perspectives provide conceptually coherent, plausible explanations for the observed prospective association between PA and PsyCap, though it must be emphasized that the present CLPM coefficients reflect group-level temporal patterns rather than confirmed individual-level causal mechanisms.

Compared with previous cross-sectional studies, the present longitudinal design the temporal evidence supporting this association (25) by demonstrating that baseline PA preceded subsequent PsyCap. Unmeasured factors, including personality characteristics, social support (23), or exercise motivation (36), may also contribute to the observed association. Future longitudinal studies with additional follow-up waves or intervention designs are warranted to further clarify the temporal dynamics and underlying mechanisms linking PA and PsyCap.

The present study further demonstrated that higher baseline PA prospectively predicted greater body appreciation at follow-up, supporting Hypothesis 2. This finding is consistent with previous studies showing that regular participation in physical activity is associated with more positive body evaluations, greater body functionality appreciation, and improved psychological wellbeing (48). Theoretically, PA may foster body appreciation by encouraging individuals to focus on what their bodies can do rather than how they look (49). However, given that the CLPM design does not statistically isolate within-person longitudinal change from stable between-person differences, this interpretation should be treated as a theoretically plausible account of group-level temporal associations rather than evidence that increasing one’s PA will necessarily produce a corresponding improvement in that person’s body appreciation.

The present findings may be particularly relevant in the context of Chinese university students. Compared with many Western populations, body-related self-evaluation in China is shaped not only by individual appearance ideals but also by collectivist cultural values, interpersonal comparison, and concerns regarding social recognition (“face”) (8). These sociocultural influences may increase vulnerability to appearance-related pressures during emerging adulthood (50). Regular participation in PA may provide opportunities to experience physical competence, enjoyment, and social interaction, thereby reducing excessive appearance-focused evaluation and promoting greater appreciation of body functionality (25, 51). The prospective association observed in this study suggests that maintaining regular PA may represent a potentially important behavioral strategy for promoting positive body image among Chinese university students (19).

Beyond the hypothesized unidirectional pathways, the cross-lagged model revealed significant reverse cross-lagged effects that warrant cautious theoretical attention. Specifically, baseline PsyCap predicted subsequent PA engagement (*β* = 0.053, *p* < 0.001), baseline body appreciation predicted subsequent PA engagement (*β* = 0.024, *p* < 0.001), and baseline body appreciation predicted subsequent PsyCap levels (*β* = 0.035, *p* = 0.004). Consistent with our earlier interpretive framing for the hypothesized forward paths, these reverse coefficients should not be interpreted as evidence that an individual’s increase in body appreciation directly drives that individual to subsequently increase their PA or PsyCap; rather, they indicate that individuals with higher baseline body appreciation tend to report higher subsequent levels of PA and PsyCap at the group level. These small, exploratory effects suggest that positive body image may function not only as a desirable outcome of health-promoting behaviors but also act as an active psychological resource that is weakly prospectively associated with future psychological adaptation and behavioral engagement (51). From the perspective of the broaden-and-build theory (29), appreciating one’s body functionality may predicted positive emotions that broaden attention and cognition, potentially co-occurring with the accumulation of psychological capital and greater engagement in health behaviors. However, the observed small effects imply that such processes, if present, are likely gradual and incremental rather than robust. Similarly, self-determination theory (12, 13, 33) would predict that individuals who appreciate their bodies for what they can do (rather than how they look) may experience greater intrinsic motivation and autonomous regulation (33), which in turn is theorized to sustain long-term PA participation. Yet the modest magnitude of the reverse paths suggests that this theoretical chain may operate weakly in the short term. Collectively, these bidirectional prospective associations provide preliminary, small-effect evidence that PA, PsyCap, and body appreciation may exhibit weak reciprocal prospective associations over time. Given the very small magnitude of these reverse paths, these observed patterns should not be interpreted as empirical support for a dynamic system or causal reciprocity; rather, they describe exploratory prospective statistical associations that can serve as a valuable reference point for the design of future high-frequency multi-wave longitudinal or controlled experimental research. Understanding these temporal associations as potentially reciprocal and co-developing may have implications for future intervention research (52). Should causal links be confirmed in future experimental or quasi-experimental research, interventions targeting any one of these constructs might indirectly influence the others through reciprocal prospective pathways, underscoring the potential value of integrated, multi-component health promotion approaches.

The observed gender disparities in PA, PsyCap, and body appreciation qualify as statistically significant descriptive findings; however, their underlying causes were not examined in the present study. Although nationwide evidence indicates that Chinese female university students report lower body appreciation than their male counterparts (53). We did not assess constructs such as appearance-related pressure, self-objectification, feminine ideals, or gender-role expectations. Consequently, explanations attributing these disparities to differential sociocultural pressures, while theoretically plausible, should be regarded as contextual hypotheses rather than empirically supported interpretations of the current data. Drawing on existing literature, one speculative possibility is that women experience greater exposure to appearance-related pressures and narrowly defined feminine ideals (8, 54), whereas male students may place relatively greater emphasis on physical competence and athletic attributes (35). If these untested assumptions are empirically supported, physical activity that centers the experiences of competence, enjoyment, and body functionality may therefore be particularly relevant to positive body evaluation among female students (53). Crucially, because appearance-ideal internalization and gender-role expectations were not measured here, these explanatory accounts explanatory remain plausible theoretical possibilities that are pending direct empirical testing.

An important finding of the present study is that different dimensions of PA demonstrated distinct dose–response relationships with PsyCap and body appreciation. It should be noted that these polynomial regression analyses primarily capture cross-sectional, between-person patterns at each assessment wave rather than intra-individual changes over time; consequently, they should not be interpreted as evidence that modifying a specific PA dimension within an individual will necessarily produce the corresponding psychological change. A key finding of the present study is that different dimensions of PA demonstrated distinct dose–response relationships with PsyCap and body appreciation. Specifically, exercise intensity showed predominantly positive linear associations, whereas exercise duration and frequency exhibited nonlinear relationships (55). These patterns are potentially consistent with the hypothesis that psychological responses to PA may exhibit threshold or saturation effects (56), whereby moderate levels of exercise duration and frequency may provide sufficient physiological stimulation and repeated opportunities for mastery, emotional regulation, and positive social interaction, thereby facilitating the development of psychological resources and more adaptive body evaluations (56). Conversely, it is plausible that insufficient PA may not provide adequate psychological stimulation, whereas excessive exercise could increase physical fatigue, recovery demands, or appearance-related pressure (16), thereby attenuating potential psychological benefits. However, these interpretations remain speculative because the proposed mechanisms—such as physiological stimulation, fatigue, recovery demands, and appearance-related pressure were not directly measured in the present study. They should therefore be treated as theoretically grounded hypotheses requiring empirical verification rather than as established explanatory mechanisms (57).

Several mechanisms may help explain these nonlinear patterns. These findings are broadly consistent with the concept that psychological responses to physical activity may exhibit threshold or saturation effects (58). Moderate levels of exercise duration and frequency may provide sufficient physiological stimulation and repeated opportunities for mastery, emotional regulation, and positive social interaction, thereby facilitating the development of psychological resources and more adaptive body evaluations. In contrast, insufficient PA may not provide adequate psychological stimulation, whereas excessive exercise could increase physical fatigue, recovery demands, or appearance-related pressure, thereby attenuating potential psychological benefits (59). These interpretations are broadly consistent with previous studies reporting U-shaped or other nonlinear associations between PA and mental health outcomes (59). Furthermore, although the nonlinear models reached statistical significance, their explanatory power was modest, particularly for PsyCap (*R*^2^ = 0.071–0.089). Given the large sample size (*n* = 669), even effects of small magnitude can achieve statistical significance, yet the proportion of variance explained by the model remains limited (60). Consequently, these observed dose–response patterns should be interpreted with considerable caution, and claims regarding the “psychological benefits” of specific PA dimensions should not be overstated. It remains unclear whether these statistically detectable patterns translate into meaningful real-world effects, and their practical implications are pending confirmation in longitudinal and intervention studies involving objective PA measurement.

The present study further demonstrated that PsyCap partially mediated the prospective association between PA and body appreciation, supporting Hypothesis 4. From a theoretical standpoint, this finding offers two tentative contributions. First, it provides longitudinal evidence consistent with the possibility that PsyCap represents one prospective statistical pathway that may account for a small portion of the covariation between PA and more positive body evaluations over time at the between-person level. However, because the CLPM does not disaggregate between-person variance from within-person change, this mediation should be interpreted as a group-level statistical pattern rather than as evidence that PA causes changes in body appreciation through PsyCap within individuals. Given the modest standardized indirect effect [*β* = 0.021, 95% CI (0.006, 0.043)], PsyCap should be regarded as one possible longitudinal correlate among several, rather than a major explanatory mechanism (25, 51). While previous research has emphasized domain-specific mechanisms—such as changes in physical self-perceptions or social physique anxiety—our results indicate that generalizable positive psychological resources (i.e., the four components of PsyCap) are prospectively associated with body appreciation in the context of PA levels that are also prospectively associated with body appreciation. This statistical pattern does not establish that PsyCap carries forward any benefits of PA; rather, it identifies one possible longitudinal correlate among several potential pathways (21). This pattern is consistent with an integrative theoretical model in which PA is hypothesized to be prospectively associated with the development of broad-based psychological capital, which is in turn hypothesized to be concurrently associated with reduced vulnerability to sociocultural appearance pressures and a stronger orientation toward functionality-based body appreciation. Second, the observation that PsyCap only partially mediates this association is theoretically consistent with a multi-pathway theoretical framework: PA may be associated with body appreciation through simultaneous yet distinct routes, including direct experiential pathways (e.g., repeated mastery of physical tasks), social pathways (e.g., peer support during exercise), and cognitive pathways (e.g., reduced self-objectification) (61, 62). Specifically, higher baseline PA was prospectively associated with greater subsequent self-efficacy, resilience, hope, and optimism, which were in turn prospectively associated with more adaptive and appreciative body evaluations. This interpretation is consistent with the broaden-and-build theory (29, 30), which proposes that positive experiences gradually build enduring psychological resources that contribute to subsequent psychological wellbeing.

Notably, although the indirect effect was statistically significant, its standardized effect size was relatively modest [standardized *β* = 0.021, 95% CI (0.006, 0.043)]. Given the small magnitude of this effect and the observational design, the clinical or practical significance of this mediation pathway should not be overstated, and PsyCap should be regarded as one potential psychological correlate among several, rather than the primary mechanism through which PA produces body appreciation. Theoretically, this modest effect size underscores the inherent complexity of the PA--body image relationship and cautions against against overly simplistic, mono-mechanistic explanations. Future theoretical models should therefore incorporate PsyCap as one component within a broader network of potential mediators, which may also include biological (e.g., neuroendocrine changes), social (e.g., belongingness), and cultural (e.g., collectivist coping styles) variables.

The present study has several notable strengths. First, by employing a two-wave longitudinal design, this study extends previous cross-sectional research and provides stronger temporal evidence for the prospective associations among PA, PsyCap, and body appreciation. Second, the application of polynomial regression enabled the identification of distinct nonlinear dose–response relationships across different dimensions of PA, providing a more comprehensive understanding than conventional linear models. Third, the integration of cross-lagged panel modeling and longitudinal mediation analysis provided additional insight into the temporal relationships and potential psychological pathways linking physical activity with body appreciation.

## 5 Limitations

First, the observational nature of this study precludes any causal conclusions. Although we adjusted for sex, age, and academic year, the models did not account for several potentially important confounders, including body mass index (BMI), social-media exposure, exercise motivation, stable personality traits (e.g., neuroticism, extraversion, conscientiousness), and baseline mental health status. Future studies should employ randomized controlled designs or statistically control for a broader range of confounders to reduce the risk of residual confounding. Second, the reliance on a two-wave design imposes specific constraints on longitudinal inference that should be carefully considered when interpreting the findings. Specifically, with only two measurement occasions, more advanced longitudinal approaches—such as the Random-Intercept Cross-Lagged Panel Model (RI-CLPM) or latent growth curve modeling—could not be implemented, as these methods typically require three or more waves to reliably separate between-person stability from within-person change (63). Critically, conventional cross-lagged panel models (CLPMs) conflate between-person differences with within-person processes; therefore, the cross-lagged coefficients reported in this study should not be interpreted as evidence that an individual’s increase in physical activity necessarily produces an individual-level increase in psychological capital or body appreciation. Rather, these coefficients reflect a mixture of stable between-person differences and intra-individual changes over time, and they establish temporal precedence and statistical covariation at the group level rather than causal mechanisms at the individual level. Consequently, the present analyses cannot disentangle stable between-person differences from intra-individual changes over time, and the observed cross-lagged coefficients may reflect a mixture of both processes. Future studies incorporating three or more assessment waves are encouraged to employ these advanced modeling techniques to better isolate within-person dynamics. Conducted for the polynomial regression models in the present study, which precluded a thorough assessment of key model assumptions, including the homogeneity of variance of residuals. Future studies should therefore employ comprehensive residual diagnostics, such as Breusch-Pagan, White, or visual inspection of residuals versus fitted values to formally evaluate heteroscedasticity and other model assumptions before interpreting nonlinear regression findings. Fourth, all variables were assessed using self-report questionnaires, which may be subject to recall and social desirability biases. Future studies could incorporate objective measures of physical activity, such as accelerometers or wearable devices, to improve measurement accuracy. Fifth, the sample consisted exclusively of Chinese university students, which may limit the generalizability of the findings to other age groups and cultural contexts. Sixth, although PsyCap partially mediated the prospective association between PA and body appreciation, the relatively modest standardized indirect effect suggests that additional mechanisms remain to be identified. Future studies should therefore examine a broader range of biological, psychological, and sociocultural pathways.

Overall, the present findings contribute to a more nuanced understanding of how distinct dimensions of PA are prospectively associated with positive body image in young adults, and they underscore the potential relevance of considering generalizable psychological resources (i.e., PsyCap) as one variable of interest when designing PA-based health promotion strategies, pending experimental confirmation that changes in PsyCap can be causally influenced by PA and that such changes produce downstream benefits for body appreciation. Furthermore, because the CLPM coefficients conflate between-person differences with within-person processes, any references to the ‘effects’ or ‘benefits’ of PA should be understood as describing group-level temporal associations rather than individual-level causal mechanisms. The primary longitudinal findings of this study are that baseline PA prospectively predicted subsequent PsyCap and body appreciation, and that PsyCap partially mediated the PA–body appreciation association. In addition, small but statistically significant reverse prospective associations were observed (*β* = 0.024–0.053). However, these reverse paths were substantially smaller than the hypothesized forward paths (*β* = 0.121–0.264) and were not hypothesized. Therefore, they should be treated as small, exploratory reciprocal associations rather than evidence of a dynamic or mutually reinforcing system. Replication using multi-wave longitudinal or experimental designs is necessary before stronger conclusions regarding reciprocal dynamics can be drawn. If causal links are subsequently established in future experimental research, interventions primarily targeting PA may indirectly influence PsyCap and body appreciation; however, given the modest effect sizes and observational nature of the current data, such reciprocal or synergistic effects should not be assumed and require empirical confirmation.

## 6 Conclusion

The present study documented significant gender differences in physical activity, psychological capital, and body appreciation, with male students reporting higher levels across all three variables. Its core longitudinal findings indicate that baseline physical activity prospectively predicted subsequent psychological capital and body appreciation, and that psychological capital partially mediated the prospective association between physical activity and body appreciation. Notably, different dimensions of physical activity demonstrated distinct dose–response relationships with these psychological outcomes, underscoring the importance of disaggregating exercise intensity, duration, and frequency rather than treating physical activity as a unitary construct. However, these dose–response patterns were characterized by modest effect sizes (*R*^2^ = 0.071–0.174), indicating that PA dimensions explain only a small proportion of the variance in PsyCap and body appreciation. Although small reverse prospective associations were also observed, their very small effect sizes (*β* = 0.024–0.053) render them exploratory, and they should not be interpreted as evidence of reciprocal or dynamic relationships. Crucially, it must be emphasized that these findings derive from an observational two-wave design and therefore establish temporal precedence and statistical association, not causality. Overall, these findings extend previous cross-sectional evidence by providing longitudinal support for the relationships among physical activity, psychological capital, and body appreciation, and may help inform the development of PA-based health promotion strategies aimed at improving both physical and psychological wellbeing among university students, pending future experimental confirmation of these prospective patterns.

## Data Availability

The raw data supporting the conclusions of this article will be made available by the authors, without undue reservation.
