# Supplementary material for: More is not always better: longitudinal associations and nonlinear dose–response relationships between physical activity, psychological capital, and positive body image among Chinese university students
Source: Front Public Health. 2026 Aug 28;14:1870988. doi: 10.3389/fpubh.2026.1870988 (PMC13561938; doi:10.3389/fpubh.2026.1870988)
Supplement: Supplementary file 1 [file Table_1.DOCX]

**Table S1** Nonparametric Mann-Whitney U test

|  | Gender | Mean Rank | Z | P25 | P75 | IQR | p |
| --- | --- | --- | --- | --- | --- | --- | --- |
| PA T1 | Male | 352.82 | -2.669 | 18.00 | 64.00 | 46 | 0.008 |
|  | Female | 312.81 |  |  |  |  |  |
| PA T2 | Male | 351.18 | -2.423 | 18.00 | 64.00 | 46 | 0.015 |
|  | Female | 314.86 |  |  |  |  |  |
| PsyCap T1 | Male | 366.91 | -4.765 | 76.00 | 104.00 | 28 | <0.001 |
|  | Female | 295.28 |  |  |  |  |  |
| PsyCap T2 | Male | 365.97 | -4.626 | 74.00 | 104.00 | 30 | <0.001 |
|  | Female | 296.44 |  |  |  |  |  |
| BA T1 | Male | 370.08 | -5.242 | 31.00 | 43.00 | 12 | <0.001 |
|  | Female | 291.33 |  |  |  |  |  |
| BA T2 | Male | 357.44 | -3.353 | 29.00 | 43.00 | 14 | <0.001 |
|  | Female | 307.06 |  |  |  |  |  |

PsyCap = Psychological capital, BA = Body Appreciation, P25 = Lower quartile ; P75 = Upper quartile, IQR‌ = ‌Interquartile range, T1 = Test 1, T2 = Test 2.

**Figure S1** Panel A presents comparisons between male and female participants. Panels B–D show age-specific distributions of physical activity, psychological capital, and body appreciation at Test 1 and Test 2.

**Table S2** Normality Test for Residuals

| Physical Activity‌ | Psychological Capital | Body Appreciation |
| --- | --- | --- |
|  | Shapiro-Wilk (*p*) | |
| Intensity | 0.114 | <0.001 |
| Duration | 0.214 | 0.001 |
| Frequency | 0.061 | 0.001 |

**Figure S2** Intensity versus psychological capital linear model Q-Q plot

**Figure S3** Duration versus psychological capital linear model Q-Q plot

**Figure S4** Frequency versus psychological capital linear model Q-Q plot

**Figure S5** Intensity versus Body Appreciation linear model Q-Q plot

**Figure S6** Duration versus Body Appreciation linear model Q-Q plot

**Figure S7** Frequency versus Body Appreciation linear model Q-Q plot
